# Differentiation of Apical Bud Cells in a Newly Developed Apical Bud Transplantation Model Using GFP Transgenic Mice as Donor

**DOI:** 10.1371/journal.pone.0150766

**Published:** 2016-03-15

**Authors:** Naoki Maruo, Ryuji Sakagami, Yasunori Yoshinaga, Kazuhiko Okamura, Yoshihiko Sawa

**Affiliations:** 1 Section of Periodontology, Department of Odontology, Fukuoka Dental College, Fukuoka, Japan; 2 Section of Pathology, Department of Morphological Biology, Fukuoka Dental College, Fukuoka, Japan; 3 Section of Functional Structure, Department of Morphological Biology, Fukuoka Dental College, Fukuoka, Japan; Seoul National University, REPUBLIC OF KOREA

## Abstract

Rodent mandibular incisors have a unique anatomical structure that allows teeth to grow throughout the lifetime of the rodent. This report presents a novel transplantation technique for studying the apical bud differentiation of rodent mandibular incisors. Incisal apical end tissue with green fluorescent protein from transgenic mouse was transplanted to wild type mice, and the development of the transplanted cells were immunohistologically observed for 12 weeks after the transplantation. Results indicate that the green fluorescent apical end tissue replaced the original tissue, and cells from the apical bud differentiated and extended toward the incisal edge direction. The immunostaining with podoplanin also showed that the characteristics of the green fluorescent tissue were identical to those of the original. The green fluorescent cells were only found in the labial side of the incisor up to 4 weeks. After 12 weeks, however, they were also found in the lingual side. Here the green fluorescent cementocyte-like cells were only present in the cementum close to the dentin surface. This study suggests that some of the cells that form the cellular cementum come from the apical tissue including the apical bud in rodent incisors.

## Introduction

Rodent maxillary and mandibular incisors have unique anatomical structures that allow the teeth to grow throughout the lifetime of the animal. There is enamel structure formed on the labial side and periodontal ligament in the lingual side. In the most apical part of the incisor, there is a specialized epithelial cell group termed an apical bud, which was originally termed as a labial cervical loop [1–3]. It has been shown that pluripotent cells and stem cell niches are present in the apical bud as well as in nails and hair follicles [4,5]. The apical bud is regarded as an analog of the cervical loop of mouse molars and human teeth in the developmental stage. This tissue is thought to be an ideal model to study epithelial-mesenchymal interactions and cell differentiation [4].

Transplantation and implantation of rodent teeth and tooth germs in the molar have shown complete tooth development [6], but it was not possible to identify any report on the transplantation of rodent mandibular incisors except for the Slavkin’s work using fertilized chicken eggs as recipients [7]. Simple extraction of mouse mandibular incisors leaves the apical soft tissue in the bone, and we planned surgically to replace the apical tissue by a fenestration procedure on the buccal surface of the mandibular bone.

Transgenic mice with green fluorescent protein (GFP) were used for providing the traceable tissue in this study. The GFP is responsible for the green bioluminescence of the jellyfish Aequorea victoria. Transgenic mice with an ‘enhanced’ GFP (EGFP) cDNA under the control of a chicken beta-actin promoter and cytomegalovirus enhancer are called ‘green mice’ that have been used for tissue transplantation and differentiation experiments. All of the tissues from these transgenic mice, with the exception of erythrocytes and hair, were green under excitation light [8].

Podoplanin is a unique cell surface marker originally found in the lymphatic vessels [9]. Sawa has reported that the podoplanin positive cells are found in the inner enamel epithelium and odontoblasts in mice [10]. Combining the findings from anti-podoplanin immnostaining and GFP enables us to distinguish the cells with specific cell characters from the green mice.

The primary purpose of this experiment is to establish a surgical apical bud transplantation and differentiation model using the green mice as donors. The apical bud and surrounding tissue were obtained from the green mice, and was used to replace the original tissue in the wild type mice. To the best of our knowledge, this is the first paper that presents the transplantation method of the rodent mandibular incisor.

The secondary purpose of this experiment is to investigate the so far unproven hypothesis that cementoblasts in the periodontal ligament are derived from epithelial precursor cells [11–15]. Some researches failed to show the evidence of the epithelial root sheath (ERS) to undergo epithelial-mesenchymal transition (EMT) during initial cellular cementogenesis [16,17]. Whether the ERS is influencing the residual mesenchymal cells to differentiate into cementoblasts, or whether sheath cells themselves can become cementoblasts through EMT is still unknown. This study was undertaken to elucidate the cell migration and differentiation of the epithelial origin cells in the apical bud of rodent incisors.

## Materials & Methods

This study was carried out in strict accordance with the recommendations in the Guide for the Care and Use of Laboratory Animals of the National Institutes of Health. The protocol was approved by the Committee on the Ethics of Animal Experiments of Fukuoka Dental College, Fukuoka, Japan (Permit Number: 12008). All surgery was performed under sodium pentobarbital anesthesia, and all efforts were made to minimize suffering.

### Transplantation

In this experiment, we used a total of 10, 5 male and 5 female, 1-week-old C57BL/6-Tg (CAG-enhanced green fluorescence protein, EGFP) C14-Y01-FM131Osb mice (green mice) as donors and a total of 10 male 4-week-old C57BL/6 mice as recipients [8,18–20]. All of the tissue from green mice, with the exception of erythrocytes and hair, were reported to be green under excitation light [8]. The green fluorescence was directly detected by exposing specimens to 488 nm laser light.

### Donor site

**Fig 1:**
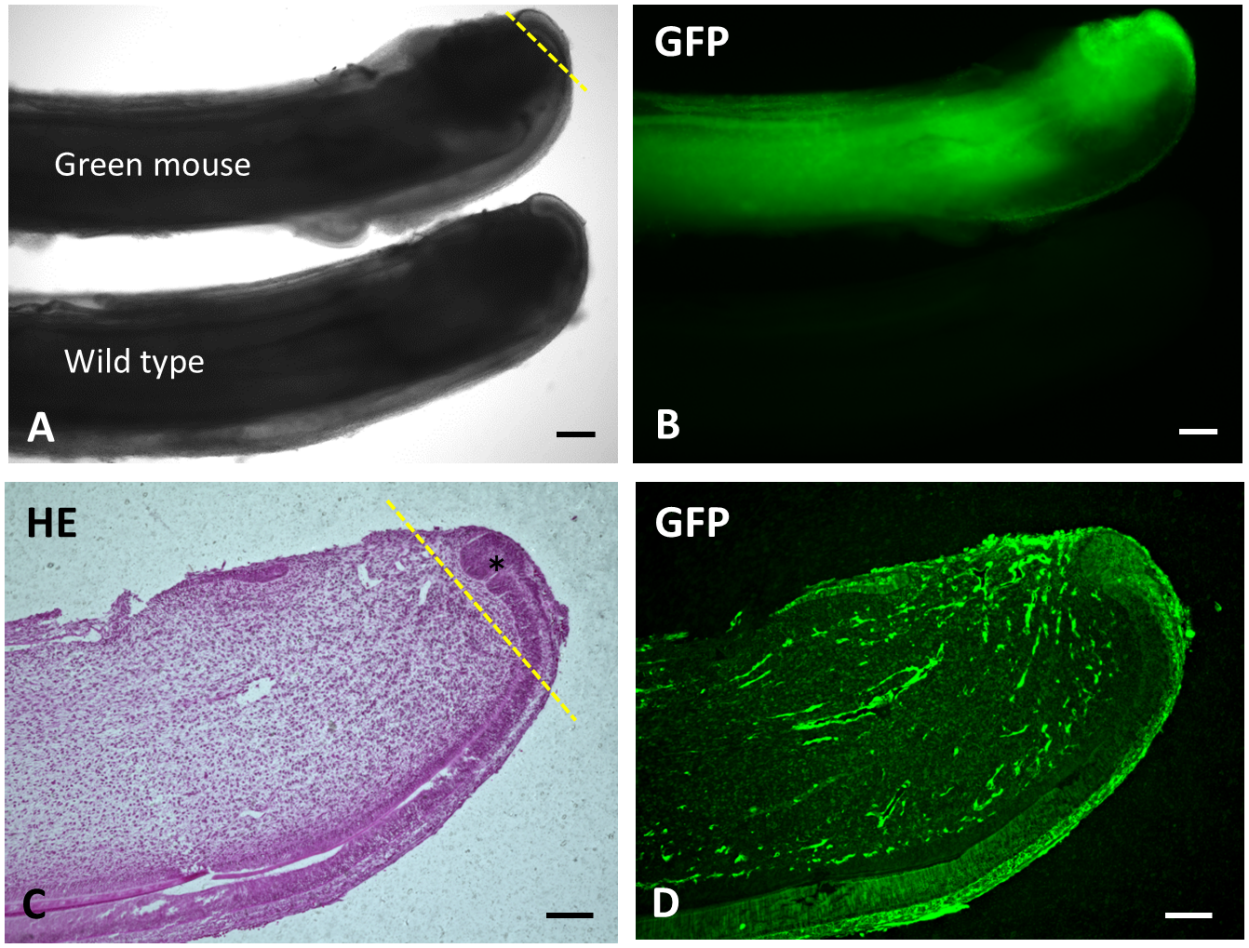
Green fluorescence expression in 1-week-old C57BL/6-Tg (CAG-EGFP) mouse mandibular incisors. (**A**) Mandibular incisors of the C57BL/6-Tg (CAG-EGFP) mouse (green mouse, top) and the C57BL/6 mouse (wild type, bottom) in phase contrast image. Incisors from the green mice showed identical shapes, sizes, and development as those from the wild type mice. The graft tissue was separated at the yellow dotted line. (**B**) Fluorescent image of the same teeth as **A**. The green fluorescence is observed only in the mandibular incisor of the green mouse. (**C**) Sagittal section stained with hematoxylin and eosin of the green mouse mandibular incisor. Asterisk indicates the apical bud. The graft tissue was separated at the yellow dotted line. (**D**) Fluorescent image of the same section as **C**. Green fluorescence is observed throughout the section with different intensities. Scale bars: 200 μm in **A** and **B**; 100 μm in **C** and **D**.

After euthanizing the green mice with isoflurane, intact incisors were dissected from both sides of the mandible, most of the dental follicle tissue surrounding the tooth apex was carefully removed with tweezers, and apical tissue containing the apical bud was separated under a stereomicroscope with 20-fold magnification. Graft tissue, 200 μm long, from the tip of the apex was stored in saline before the transplantation (Fig 1).

### Recipient site

Wild type mice were anesthetized with 50 mg/kg pentobarbital. After local administration of 0.2% xylocaine with 1/800,000 epinephrine to the mandible, a 10 mm long incision was made in the skin, and the masseter muscle was cut at the level of the occlusal plane. The buccal bone around the tooth apex was fenestrated with a #11 scalpel blade and the apical tissue was removed.

The green fluorescent tissue was transplanted to the location of the removed original tissue and the bony hole was covered with a Gore-Tex^®^ membrane. The masseter muscle and the skin were respectively closed with an absorbable suture. The experimental mice were returned to their individual cages and allowed to move freely. The mice were fed with normal diet during the healing period and carefully monitored everyday.

### Tissue preparation

A total of 4, 4, and 2 recipient mice were sacrificed at 2 weeks, 4 weeks, and 12 weeks after the transplantation, respectively. Under administration of 50 mg/kg pentobarbital, the mice were perfused with saline followed by 1.5% paraformaldehyde phosphate buffer solution for fixation [21]. Next the hemi-mandibles of the mice were embedded in super cryoembedding medium (SCEM), and rapidly frozen using liquid N_2_. Samples were cut with a cryostat (Leica Microsystems, Wetzlar, Germany) into 4 μm sections for Hematoxylin and Eosin staining and for immunofluorescence staining. Each specimen was cut with a tungsten carbide blade without chemical decalcification using the film transfer method [22,23].

### Immunostaining

Sections were fixed in 4% paraformaldehyde phosphate buffer solution for 3 minutes at room temperature (RT), and treated with 1% goat serum (GS) diluted with 10 mM phosphate-buffered saline (PBS, pH 7.4) for 30 minutes at RT and were exposed by a primary antibody: 1 μg/ml of hamster anti-mouse podoplanin IgG (AngioBio Co., Del Mar, CA, USA) for 12 hours at 4°C. After treatment with the primary antibody, sections were reacted with a second antibody: 1 μg/ml of Alexa Fluor 568-conjugated goat anti-hamster IgG (Molecular Probes, Invitrogen, Eugene, OR) in GS-PBS for 1 hour at RT, and then examined by fluorescence microscopy, BZ-9000 (Keyence Corp., Osaka, Japan) and laser-scanning confocal microscopy (Axiovert 135M, Carl Zeiss, Jena, Germany), with a ×63 Plan-Apochromatic oil immersion objective lens (numerical aperture ×1.4) [23]. The green fluorescence was directly detected without any antibody by exposing specimens to 488 nm laser light. At the time of the embedding, DAPI (4', 6-diamidino-2-phenylindole) was counterstained for detecting regions in the DNA.

## Results

Successful transplantation and normal tooth growth was observed in 3 of 4, 2 of 4, and 1 of 2 samples at 2, 4, and 12 weeks after surgery, respectively. Of the remaining 4 samples, 2 showed ectopic green fluorescent tooth growth and 2 showed no signs of green fluorescent cells in the mandible.

### Frozen sections at 2 weeks after transplantation

**Fig 2:**
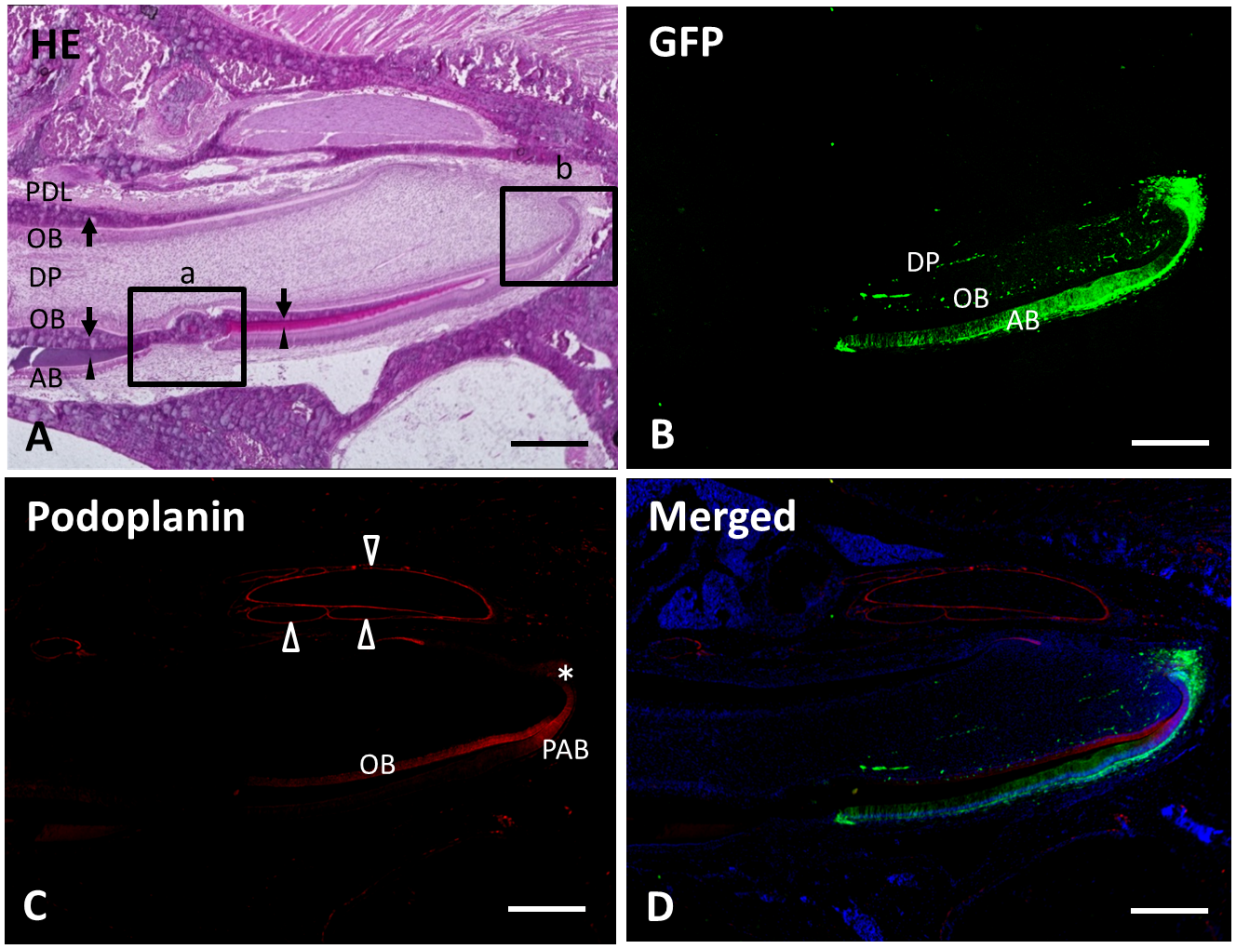
Frozen undecalcified sagittal section of the mandible 2 weeks after transplantation. The top, bottom, left, and right of each panel show the lingual, labial, incisal end, and apical end sides, respectively. (**A**) In the section stained with hematoxylin and eosin, the lingual side of the incisor presents periodontal ligament (PDL), dentin (arrow), and odontoblasts (OB). The labial tissue consists of odontoblasts (OB), dentin (arrow), enamel (arrowhead), and ameloblasts (AB). DP, dental pulp cells. Boxed areas (a) and (b) are magnified in Figs 3 and 4, respectively. (**B**) Green fluorescent cells are present at the labial half of the mandibular incisor. Green fluorescence is observed in ameloblasts (AB), odontoblasts (OB), and dental pulp cells (DP). (**C**) Reaction products with anti-podoplanin are observed in apical bud (asterisk), pre-ameloblasts (PAB), odontoblasts (OB), and nerve sheaths (outlined arrowheads). (**D**) Merged image of **B**, **C**, and blue fluorescence of DAPI’s. Scale bars: 500 μm.

At 2 weeks after surgery, transplanted green fluorescent cells were engrafted and had migrated approximately 3 mm toward the incisal edge (Fig 2). In the labial side of the incisor, the recipient and donor-derived structures are seen in the incisal end and apical end sides, respectively. The green fluorescent cells were only present at the labial half of the mandibular incisor, and green fluorescence was observed in ameloblasts, odontoblasts, and in the dental pulp cells. In the dental pulp, there is green fluorescence in the capillaries. Reaction products with anti-podoplanin were observed in apical buds, pre-ameloblasts, odontoblasts, and the nerve sheaths (Fig 2).

**Fig 3:**
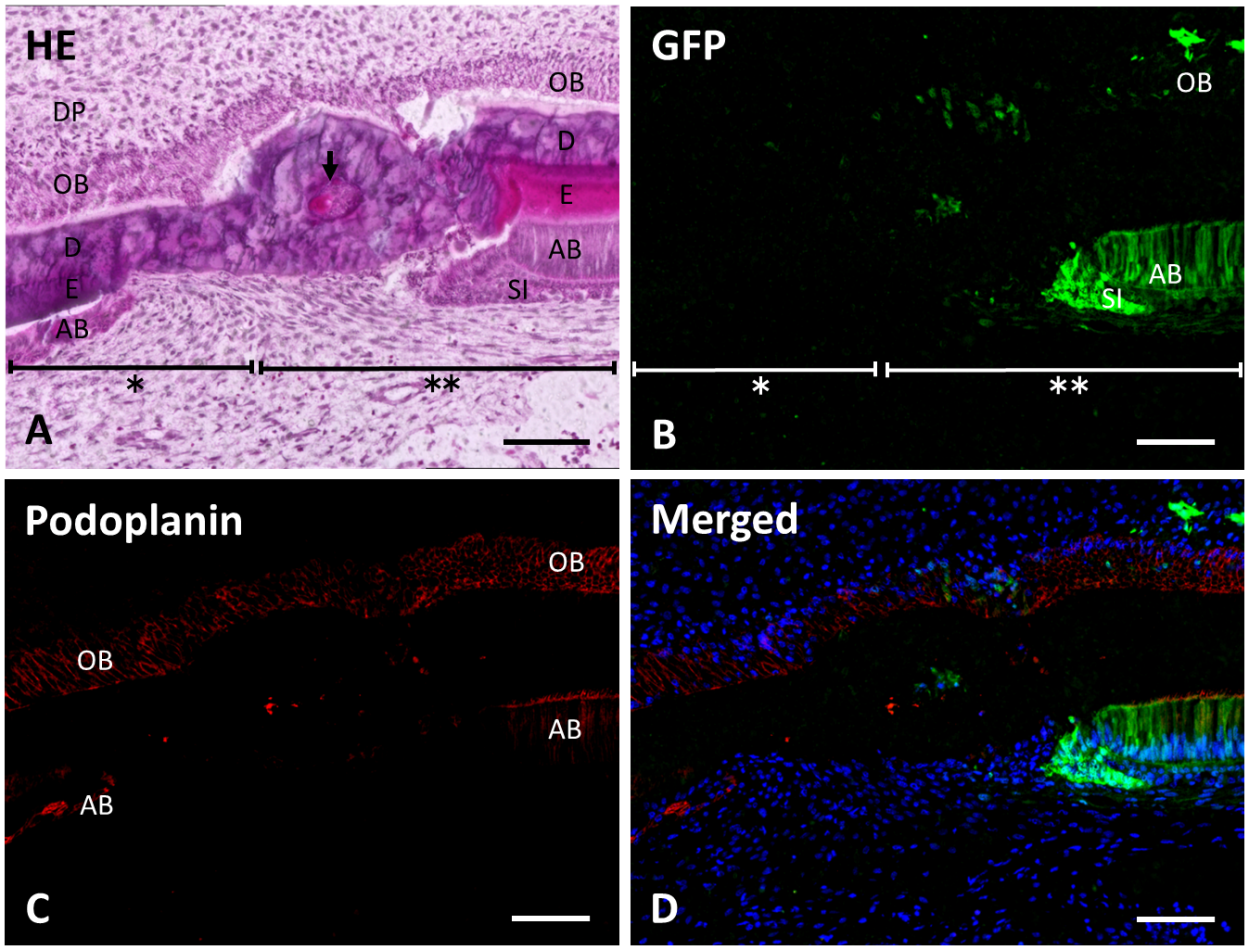
Green fluorescence in the transitional area between recipient tissue and donor derived tissue. Figures are the area of (a) in Fig 2 here at medium magnification. (**A**) In the section stained with hematoxylin and eosin, the recipient tissue (asterisk) is replaced by the donor-derived tissue (double asterisk). Ameloblasts (AB) and enamel (E) are observed both in the recipient and donor derived tissue. In the transitional area enamel-like hard tissue (arrow) is found surrounded by the dentin. Odontoblasts (OB) and dentin (D) are continuous through recipient tissue to donor derived tissue. SI, stratum intermedium. (**B**) Green fluorescence is observed in odontoblasts (OB), ameloblasts (AB), and the stratum intermedium (SI) of the donor derived tissue (double asterisk). The green fluorescence is not observed in the recipient tissue (asterisk). (**C**) Reaction products with anti-podoplanin antibody are observed in the odontoblasts (OB) and ameloblasts (AB) of the recipient and the donor derived tissue. (**D**) Merged image of **B**, **C**, and blue fluorescence of DAPI’s. Scale bars: 100 μm.

In the medium magnification the area of (a) in Fig 2, there is a transitional area between the recipient tissue and the donor-derived tissue (Fig 3). In the incisal end side, the original recipient tissue remained without any green fluorescence, and in the apical end side there were green fluorescent ameloblasts, odontoblasts, and stratum intermedium with irregular enamel-like hard tissue surrounded by dentin locating between the two areas. Odontoblasts and dentin were continuous through recipient tissue to donor-derived tissue. Ameloblasts and enamel structure were observed both in the recipient and donor derived tissue. Reaction products with anti-podoplanin antibody were observed in the odontoblasts and the ameloblasts. Podoplanin-positive odontoblasts were observed to extend continuously from the recipient tissue to the donor derived tissue (Fig 3).

**Fig 4:**
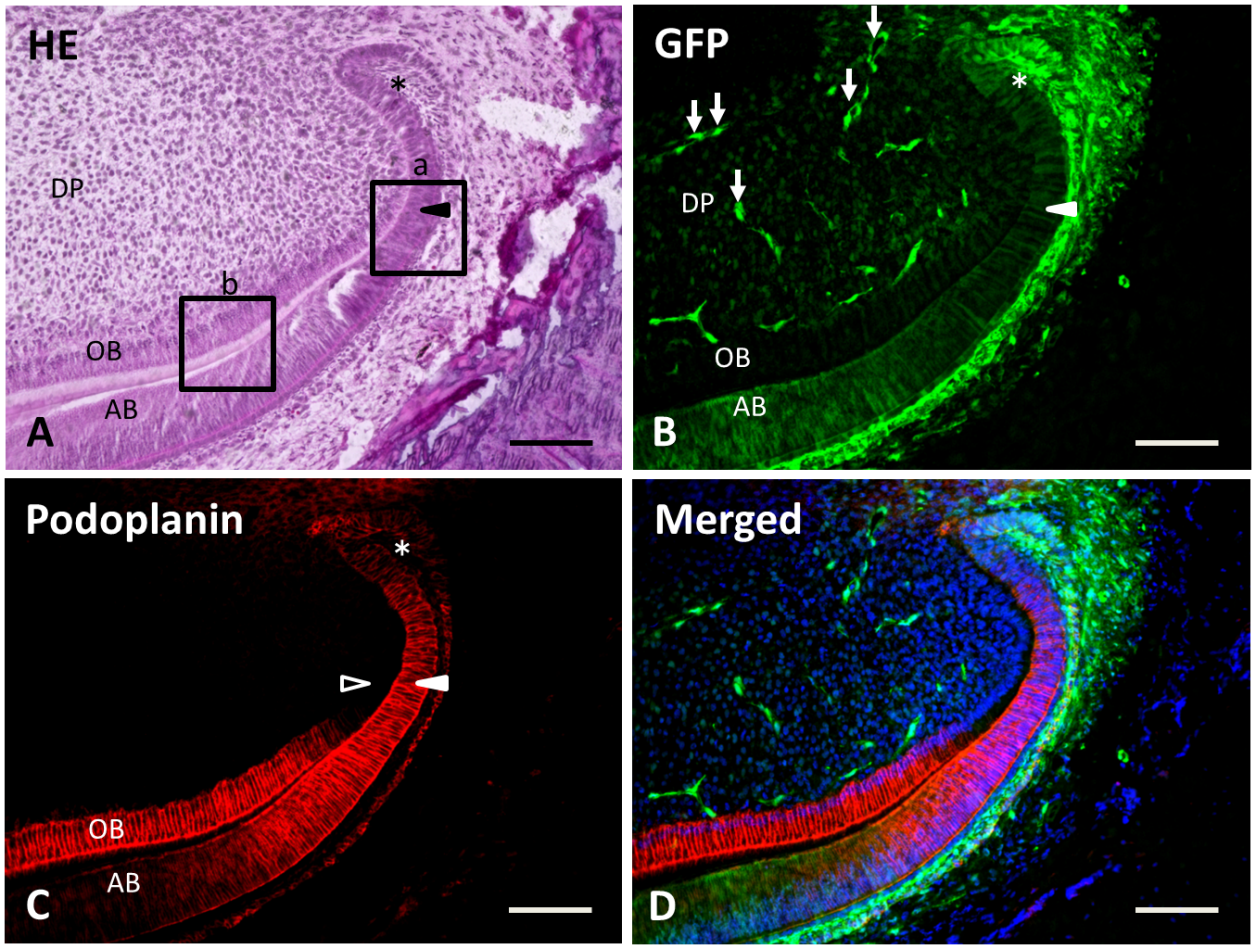
Green fluorescence and podoplanin expression in the apical end of the mandibular incisor. Figures are the area of (b) in Fig 2 here at medium magnification. (**A**) In the section stained with hematoxylin and eosin, an apical bud (asterisk), pre-ameloblasts (arrowhead), dental pulp cells (DP), odontoblasts (OB), and ameloblasts (AB) are observed. Boxed areas (a) and (b) are magnified in Figs 5 and 6, respectively. (**B**) Green fluorescence is observed in the apical bud (asterisk), pre-ameloblasts (arrowhead), ameloblasts (AB), odontoblasts (OB), and dental pulp cells (DP). Green fluorescence is found in the capillaries of the dental pulp (arrows) as well. (**C**) Podoplanin expression is observed in apical bud (asterisk), pre-ameloblasts (arrowhead), and odontoblasts (OB). Immunoreaction with anti-podoplanin is weaker in ameloblasts (AB) than in pre-ameloblasts (arrowhead), and is stronger in odontoblasts (OB) than in pre-odontoblasts (outlined arrowhead). (**D**) Merged image of **B**, **C**, and blue fluorescence of DAPI’s. Scale bars: 100 μm.

In the apical end of the incisor, the merged green fluorescent tissue showed a continuous structure without any disruptions. The dental papilla showed relatively dense cells lining the inner enamel epithelium, with the inner enamel epithelium extended forward to the pre-ameloblasts, which later become ameloblasts (Fig 4). Strong green fluorescence was observed in epithelial-origin cells from the apical buds, with moderate green fluorescence in odontoblasts and in dental pulp cells (Fig 4). Reaction products with anti-podoplanin were observed in the apical buds, pre-ameloblasts, and odontoblasts. The immunoreaction with anti-podoplanin was weaker in ameloblasts than in pre-ameloblasts. Podoplanin expression was identified in differentiated odontoblasts, but not in pre-odontoblasts (Fig 4).

**Fig 5:**
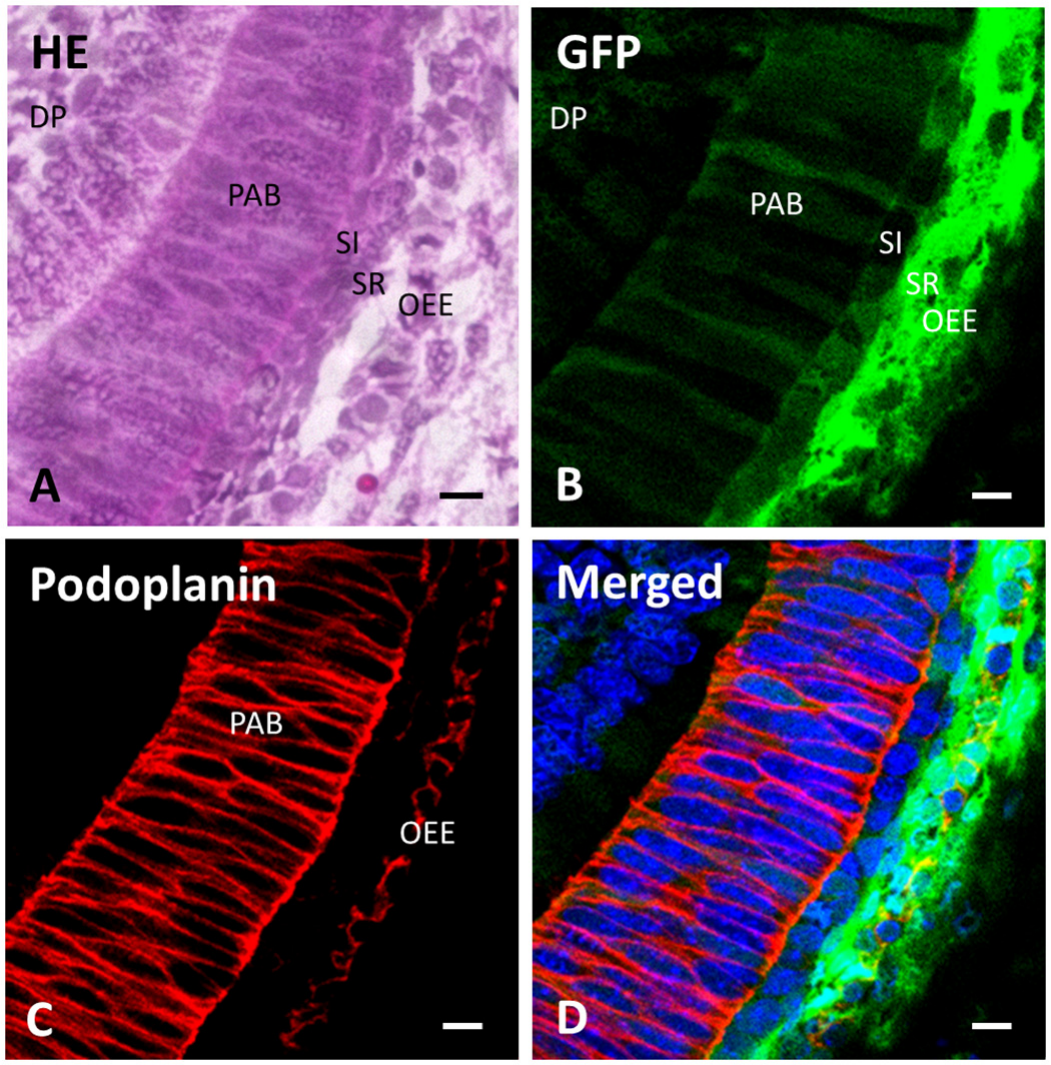
Green fluorescence and podoplanin expression in pre-ameloblasts. Figures are the area of (a) in Fig 4 at a high magnification. (**A**) In the section stained with hematoxylin and eosin, dental papilla cells (DP), pre-ameloblasts (PAB), stratum intermedium (SI), stellate reticulum (SR), and outer enamel epithelium (OEE) are observed. (**B**) Green fluorescence is observed in pre-ameloblasts (PAB), stratum intermedium (SI), stellate reticulum (SR), outer enamel epithelium (OEE) and dental papilla cells (DP). (**C**) Podoplanin expression is observed in pre-ameloblasts (PAB) and outer enamel epithelium (OEE). (**D**) Merged image of **B**, **C**, and blue fluorescence of DAPI’s. Scale bars: 10 μm.

In the observations with higher magnification at (a) of Fig 4, there are pre-ameloblasts with relatively large nuclei in 2 or 3 layers (Fig 5). The green fluorescence and podoplanin expression was observed in pre-ameloblasts and the outer enamel epithelium. Green fluorescence was also observed in stellate reticulum, stratum intermedium, and dental pulp cells (Fig 5).

**Fig 6:**
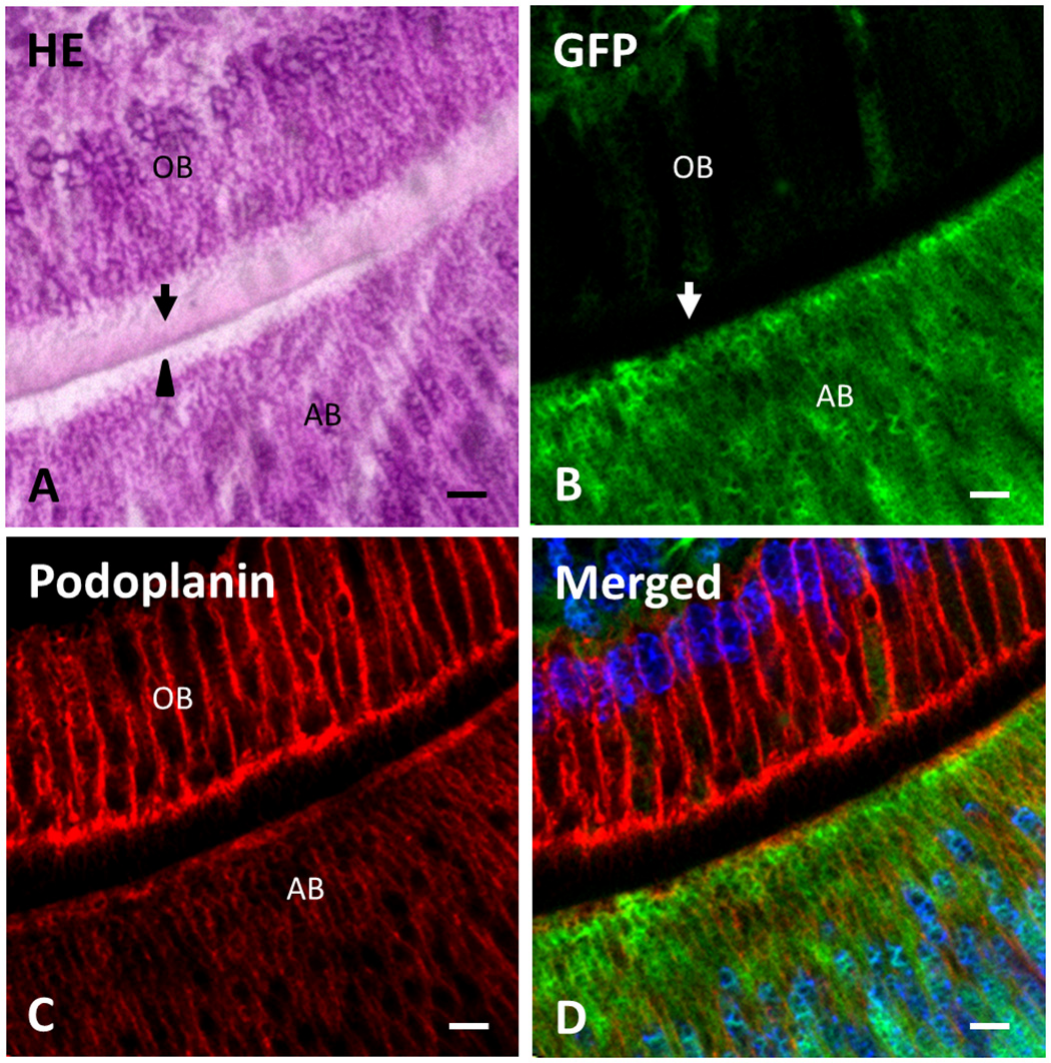
Green fluorescence and podoplanin expression in the odontoblasts. Figures are the area of (b) in Fig 4 at a high magnification. (**A**) In the section stained with hematoxylin and eosin, the formation of pre-dentin (arrow) by odontoblasts (OB) followed by the early stage enamel (arrowhead) production from ameloblasts (AB) is observed. (**B**) Green fluorescence in the odontoblasts (OB) is weaker than that in the ameloblasts (AB), but within the detectable level. Green fluorescence is minimal in the pre-dentin (arrow). (**C**) Reaction products with anti-podoplanin antibody are clearly observed in the odontoblasts (OB) and weakly observed in the ameloblasts (AB). (**D**) Merged image of **B**, **C**, and blue fluorescence of DAPI’s. Nuclei of odontoblasts (OB) are only located in the pulp side of the cells. Scale bars: 10 μm.

In the observations with higher magnification at (b) in Fig 4, the odontoblasts showed high polarity with the nuclei aligned at the dental pulp side (Fig 6). The green fluorescence was observed in odontoblasts but it was weaker than that in the ameloblasts and dental pulp cells. The green fluorescence was not observed within the matrix of newly formed pre-dentin (Fig 6). Podoplanin expression was strong in dentin forming odontoblasts and moderate in enamel forming ameloblasts (Fig 6).

### Frozen sections at 4 weeks after transplantation

**Fig 7:**
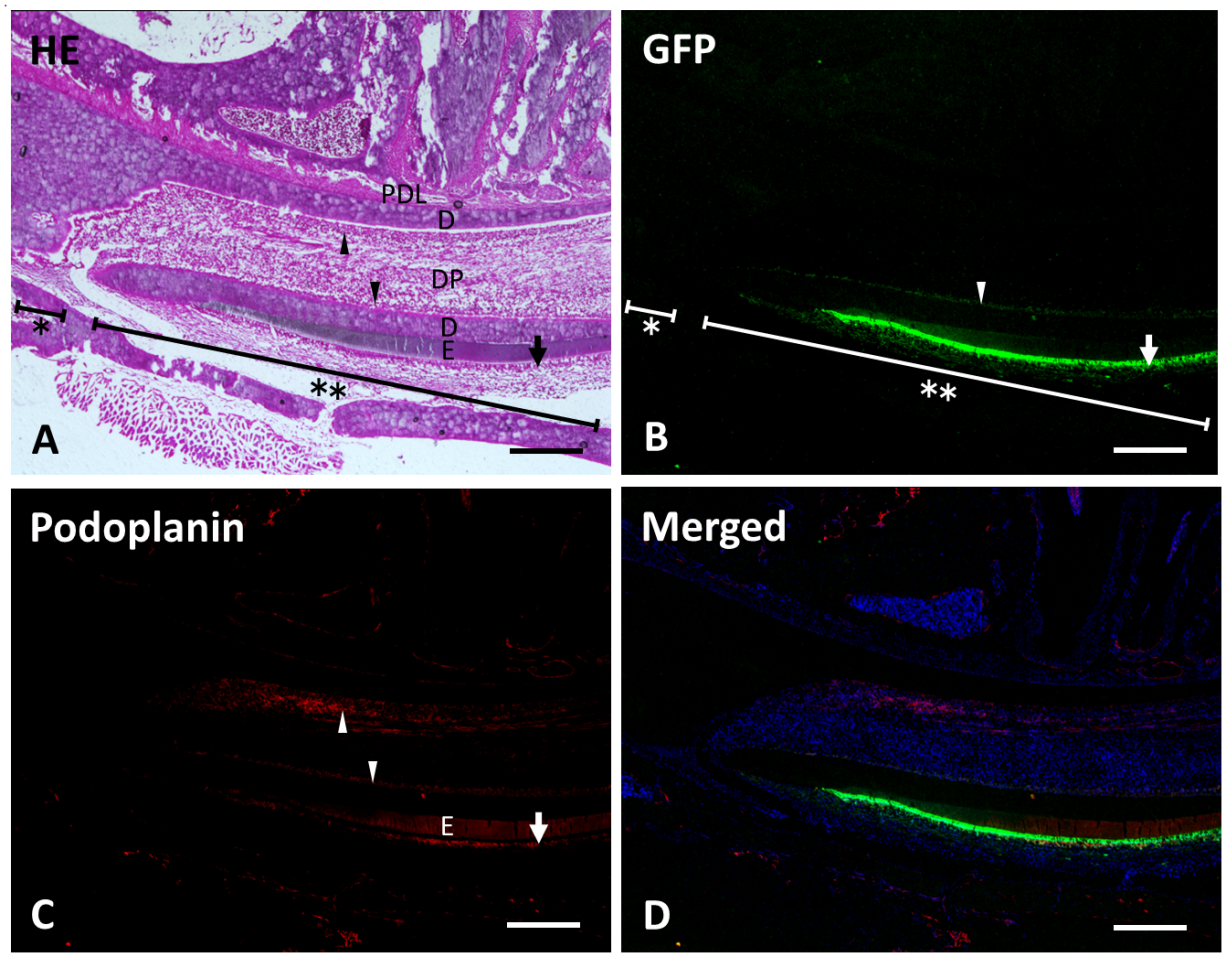
Frozen undecalcified sagittal section of a mandible 4 weeks after transplantation. The top, bottom, left, and right of each panel show the lingual, labial, incisal end, and apical end sides, respectively. (**A**) In the section stained with hematoxylin and eosin, the recipient tissue (asterisk) and donor-derived tissue (double asterisk) are observed in the labial side of the incisor. The lingual side of the incisor presents periodontal ligament (PDL), dentin (D), and odontoblasts (arrowhead). The labial tissue consists of odontoblasts (arrowhead), dentin (D), enamel (E), and ameloblasts (arrow). DP, dental pulp cells. (**B**) Green fluorescence is observed in odontoblasts (arrowhead) and ameloblasts (arrow) of the donor derived tissue (double asterisk). The green fluorescence is not observed in the recipient tissue (asterisk). (**C**) Reaction products with anti-podoplanin antibody are observed in the labial and lingual odontoblasts (arrowheads) and in the ameloblasts (arrow). There is a cross reaction with anti-podoplanin at the enamel (E). (**D**) Merged image of **B**, **C**, and blue fluorescence of DAPI’s. Scale bars: 500 μm.

**Fig 8:**
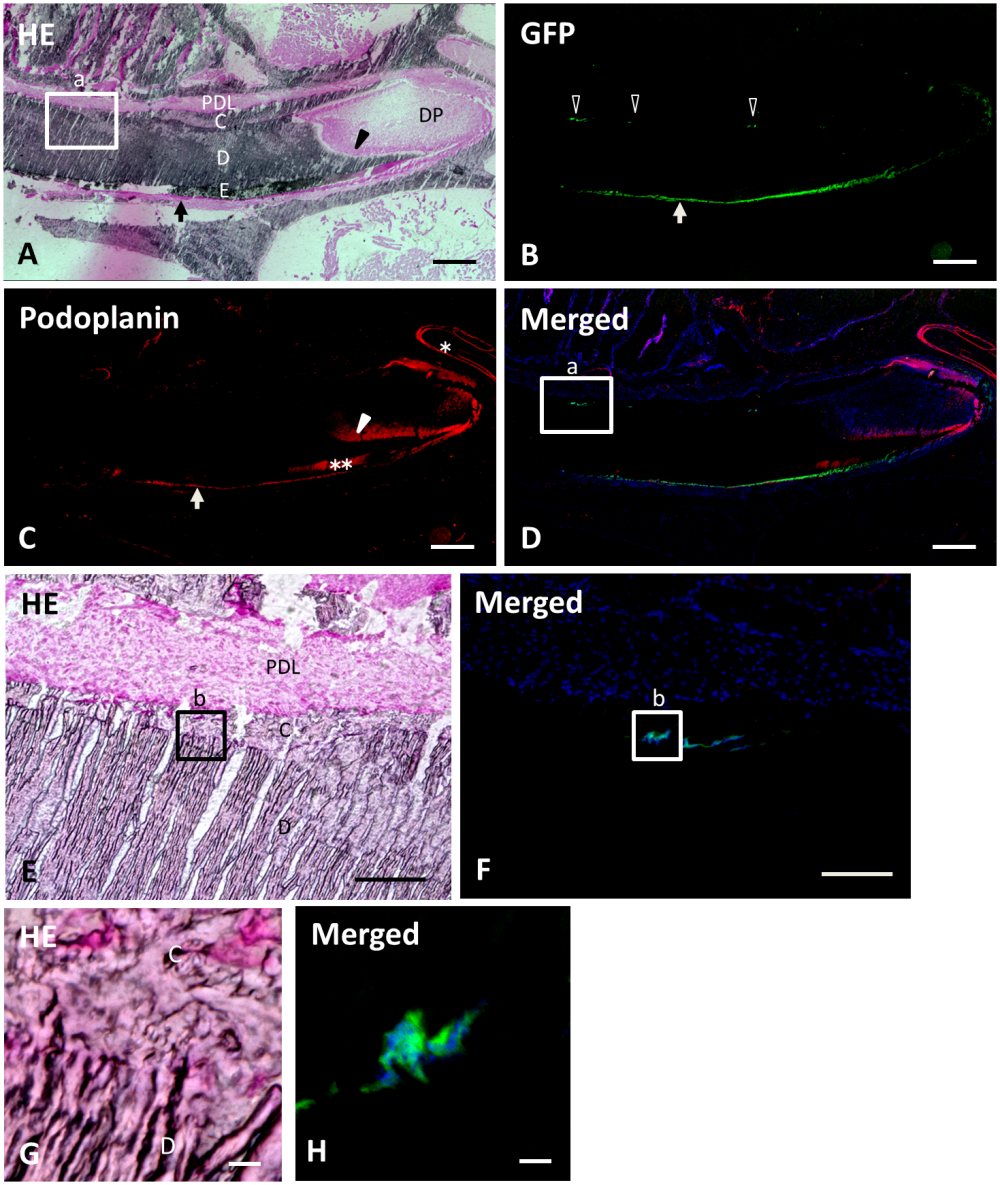
Frozen undecalcified sagittal section of mandible 12 weeks after transplantation. The top, bottom, left, and right of each panel show the lingual, labial, incisal end, and apical end sides, respectively. (**A**) In the section stained with hematoxylin and eosin, the lingual side of the incisor presents periodontal ligament (PDL), cementum (C), dentin (D), and odontoblasts (arrowhead). The labial tissue consists of odontoblasts (arrowhead), dentin (D), enamel (E), and ameloblasts (arrow). DP, dental pulp cells. (**B**) The panel shows migration of the donor derived cells with green fluorescence. There are green fluorescent cells in the lingual side (outlined arrowheads) as well as in the labial side (arrow). (**C**) Reaction products with anti-podoplanin are observed in the nerve sheaths (asterisk), odontoblasts (arrowhead), and ameloblasts (arrow). There is a location showing cross-reaction with anti-podoplanin at the enamel matrix (double asterisk). (**D**) Merged image of **B**, **C**, and blue fluorescence of DAPI’s. Boxed area (a) in **D** indicates the identical location and size of that in **A**. (**E**, **F**) Boxed area (a) of **A** and **D** at a medium magnification. (**E**) Periodontal ligament (PDL), cementum (C), and dentin (D) are observed. (**F**) Green fluorescent cells are observed in the cementum but not in the periodontal ligament. The boxed area (b) in **F** indicates the identical location and size of that in **E**. (**G**, **H**) Boxed area (b) of **E** and **F** at a high magnification, respectively. (**G**) The HE section shows cementocyte-like cells in the cementum (C) close to the dentin (D) surface. (**H**) Green fluorescent cementocyte-like cells are observed. The nuclei are observed in the green fluorescent cells. Scale bars: 500 μm in **A**, **B**, **C**, **D**; 100 μm in **E**, **F**; 10 μm in **G**, **H**.

In the frozen sections of the mandible at 4 weeks after transplantation, the recipient and donor-derived tissue were locally observed in the labial side of the incisor. The transplanted green fluorescent cells had migrated 3–4 mm further toward the incisal edge, compared to the 2 week samples (Fig 7). Strong green fluorescence expression in the ameloblasts and moderate expression in the odontoblasts was observed. Green fluorescence was not observed in the lingual side of the incisor. Reaction products with anti-podoplanin antibodies were observed in the labial and lingual odontoblasts of the incisor and in the ameloblasts (Fig 8).

### Frozen sections at 12 weeks after transplantation

In the frozen sections of the mandible 12 weeks after transplantation, the donor-derived cells were observed extending all the way to the incisal end. There were periodontal ligament, cementum, dentin, enamel, and ameloblasts from the lingual side to the labial side of the incisor. Odontoblasts and dental pulp cells were found in the dental pulp space. Reaction products with anti-podoplanin were observed in nerve sheaths, apical buds, odontoblasts, and ameloblasts. Green fluorescent cells were observed in the lingual side as well as in the labial side (Fig 8B). In the lingual side, green fluorescent cells were observed only in the cementum and no green fluorescent cells were found in the periodontal ligament space (Fig 8E and 8F). In the high magnification, green fluorescent cementocyte-like cells with nuclei were observed located in the cementum (Fig 8G–8I).

## Discussion

To establish an experimental model suitable for studying details of tooth development, we have focused on the mouse incisor that grows throughout the lifetime of mice. The green fluorescent mandibular incisors were obtained from transgenic mice. Without any preceding treatment, the green fluorescent cells from donor mice were proven to be utilized for tracing after transplantation [8,18–20,24,25]. The green fluorescence was directly detected by exposing specimens to 488 nm laser light. The green fluorescence intensity varied by cell type, i.e., stronger expression in the apical bud cells than in the pre-ameloblasts and pre-odontoblasts (Fig 1D). It seemed cells with larger proportion of nuclei in cytoplasm and/or less actin forming cells showed less GFP expression because the green mice were with an enhanced GFP (EGFP) cDNA under the control of a chicken beta-actin promoter [8].

In this experiments, immunofluorescent staining against podoplanin was performed to understand the characteristics of the replaced and differentiated cells. Podoplanin is a unique cell surface marker originally found in the lymphatic vessels [9]. Sawa has reported that the podoplanin positive cells are found in the inner enamel epithelium and odontoblasts in mice incisors [10]. Combining the findings from anti-podoplanin immnostaining and HE staining enabled us to distinguish the cells with specific cell characters such as ameloblasts, odontoblasts, and apical bud cells.

A unique surgical technique was originally developed by applying the knowledge of the periodontal surgery. The key to the successful transplantation was minimally traumatic surgery to the recipient. Bleeding during the surgery was minimal as long as the large blood vessel in the muscle and in the tooth apex was not damaged. Gore-Tex^®^ membrane was used to prevent other soft tissue from migrating into the transplantation site. Gore-Tex^®^ is a synthetic nonresorbable material frequently used in periodontal regenerative procedures.

In this experiment, 200-μm long apical tissue was used for the transplantation. Dental follicle cells from the extracted donor teeth were microscopically removed as much as possible from the apex of the tooth (Fig 1), but there was a possibility of remaining tiny amount of dental follicle cells in the donor tissue. Further study is now underway to attempt the transplantation of enzymatically isolated apical buds. We expect to be able to observe more specific details of cell development with this new approach.

The GFP is water soluble [21], and all samples had to be pre-fixed with paraformaldehyde phosphate buffer solution at the time of sacrifice. After the tissue was embedded in the compound, Kawamoto’s film method with a tungsten carbide blade was used for the sectioning so that intact hard and soft tissue could be observed without any decalcification process [22].

In the 2-week specimens, green fluorescent cells extended 3 mm from the apex of the tooth (Fig 2). Green fluorescence was observed in ameloblasts, odontoblasts, and the capillaries of the dental pulp (Fig 4). The pre-ameloblasts that located between the inner enamel epithelium and ameloblasts showed one of the most proliferated cell areas of these incisors [26] (Fig 5).

The podoplanin positive cells were found in the apical bud, pre-ameloblasts, nerve sheaths, and odontoblasts at 2 weeks. All of these cells and structures were in the same locations as in the wild type mice [9,10,27]. Considering the characteristics of the podoplanin expression in the green fluorescent cells, the ameloblasts, odontoblasts, and dental pulp cells were at the normal state and had differentiated from the transplanted apical end tissue. Therefore, the transplanted tissue was considered to be the group of pluripotent cells that had been engrafted and was differentiated in the recipient tissue. These findings concluded that this novel apical bud differentiation model using transgenic rodent incisor was successful.

The donor derived green fluorescent tissue was clearly identified in the labial side of the 2-week samples and a transitional area was observed between the green fluorescent negative and positive tissue (Fig 3). In the incisal side, the original recipient tissue remained without any green fluorescence. In the apical side, there were green fluorescent ameloblasts and odontoblasts. The ameloblasts were observed in separate locations of the recipient and donor derived tissue, whereas aligned podoplanin-positive odontoblasts appeared to be transitioning, changing from green fluorescent negative to positive (Fig 3).

In the 4-week samples, the green fluorescent cells had migrated further towards the incisal edge direction (Fig 7). The green fluorescent cells that had advanced most were located in the area below the 3rd molar at 2 weeks, and were past the area below the 1st molar at 4 weeks (Figs 2 and 7), which could be explained by the gradual eruption process of the incisor. At 4 weeks, the green fluorescent cells were only observed in the labial side of the teeth and not in the lingual side.

In the 12-week tissue samples, the cryosection was not cut satisfactorily like the 2 and 4-week samples due to the higher calcification of the tooth and bone. The thickness of the hard tissue sections seemed not to be uniform, but it was still possible to enable the observations of the dental pulp and the periodontal tissue properly (Fig 8).

The green fluorescent cells were found both in the labial side and in the lingual side of the incisors (Fig 8B). This condition was only observed in the 12-week sample and not in the 2 or 4-week samples. It may be that 4 weeks is not long enough for the labial cells to migrate to the lingual area, suggesting that the 12-week interval after the transplantation was needed for determining the characteristics of the cells migrating to the lingual side of the mandibular incisor.

In the 12-week samples, the lingual green fluorescent cells were entrapped inside the cementum close to the dentin. They located only in the cellular cementum, and were not found in the area close to or inside the periodontal ligament. Whether these green fluorescent cementocyte-like cells are cementoid-secreting cementoblasts/cementocytes or entrapped ERS is still unknown. Molecular markers specific to cementoblasts/cementocytes need to be applied to the specimen for gaining a further understanding. There is however a high probability that those cells are cementoblasts/cementocytes rather than entrapped ERS because they are located in the calcified cementum where ordinary epithelial cells are hard to survive with low-oxygen and low nutrient.

Whether these cementocyte-like cells are of epithelial or mesenchymal origin is still unknown. A part of dental follicle cells at the tooth apex was included in the donor-derived tissue. There is however the possibility that these cementocyte-like cells are of epithelial origin, because green fluorescent epithelial-related cells were the only cells extended to the incisal ends in the 12-week specimen. In mouse incisors, labial epithelial cells migrate to lingual side and become ERS [4]. The specimens needed to be cut in the transverse plane, perpendicular to the incisal axis, for elucidating this cell migration process.

In human teeth, ERS plays a significant role both in induction of the root dentinogenesis as well as the cementogenesis and the development of the periodontal ligament. During root development, the secreted products of ERS induce adjacent cells of the dental follicle to differentiate and produce new cementum. There is also the possibility that EMT takes place at ERS cells and that a part of the ERS cells themselves become cementoblasts [11–15].

The study here suggests that a part of the cells that make up the cellular cementum originates in the apical tissue including the apical bud in rodent incisors. The experimental design reported here suffers from the limitation of animal specific differences between mice and humans. To further elucidate the mechanism of the tooth development, a further study is underway with more specific cell transplantation and the application of epithelial/mesenchymal cell markers in the immunostaining.

## Conclusions

Incisal apical end tissue with GFP transgenic mouse was successfully transplanted to wild type mice, and the development of the transplanted cells toward the incisal edge direction were immunohistologically observed. After 12 weeks the green fluorescent cementocyte-like cells were found in the cementum close to the dentin surface. This study suggests that some of the cells that form the cellular cementum come from the apical tissue including the apical bud in rodent incisors.
